# Identification of a General Odorant Receptor for Repellents in the Asian Corn Borer *Ostrinia furnacalis*

**DOI:** 10.3389/fphys.2020.00176

**Published:** 2020-03-13

**Authors:** Jie Yu, Bin Yang, Yajun Chang, Yu Zhang, Guirong Wang

**Affiliations:** ^1^State Key Laboratory for Biology of Plant Diseases and Insect Pests, Institute of Plant Protection, Chinese Academy of Agricultural Sciences, Beijing, China; ^2^College of Plant Protection, Henan Agricultural University, Zhengzhou, China; ^3^Research Center for Grassland Entomology, Inner Mongolia Agricultural University, Hohhot, China; ^4^Lingnan Guangdong Laboratory of Modern Agriculture, Genome Analysis Laboratory of the Ministry of Agriculture, Agricultural Genomics Institute at Shenzhen, Chinese Academy of Agricultural Sciences, Shenzhen, China

**Keywords:** odorant receptor, heterologous expression system, host plant volatile, repellent, nonanal

## Abstract

Attractants and repellents are considered to be an environment-friendly approach for pest management. Odorant receptors (ORs), which are located on the dendritic membranes of olfactory sensory neurons in insects, are essential genes for recognizing attractants and repellents. In the Asian corn borer, *Ostrinia furnacalis*, ORs that respond to sex pheromones have been characterized, but general ORs for plant odorants, especially for repellents, have not been identified. Nonanal is a plant volatile of maize that could result in avoidance of the oviposition process for female adults in *O. furnacalis.* In this study, we identified a female-biased OR that responds to nonanal using a *Xenopus* oocyte expression system. In addition, we found that OfurOR27 was also sensitive to two other compounds, octanal and 1-octanol. Behavioral analysis showed that octanal and 1-octanol also caused female avoidance of oviposition. Our results indicated that *OfurOR27* is an OR that is sensitive to repellents. Moreover, the two newly identified repellents may help to develop a chemical ecology approach for pest control in *O. furnacalis*.

## Introduction

Chemical ecology is now established as an approach for pest management. Pheromones have been identified in many insects and are great attractants to interfere with mating behaviors by trapping large numbers of male adults (Witzgall et al., 2010). However, pheromones only attract males, which is a major limitation, because females lay eggs and the mating rate might not be affected if the males can mate many times, as in some species. In this case, plant volatiles are considered to have great application prospects since they were effective for trapping both males and females (Gregg et al., 2010). Some plant volatiles have been identified as involved in oviposition, although the perception mechanism for those plant volatiles was still unknown (Mitchell et al., 1990; Koul et al., 2013). Studies on this topic should help to develop chemical ecology methods for pest management.

Insects have evolved a complex olfactory system to detect various odorants to search for mating partners, locate host plants, identify oviposition sites, and evade toxicants and predators (Bruce et al., 2005; Bruyne and Baker, 2008; Pelletier et al., 2015; Gadenne et al., 2016). Antennae of insects are the main organs for chemoreception and function directly in the process of sensing environmental information. On the antennae, a variety of sensilla are distributed, which usually contain two or more olfactory sensory neurons (OSNs) inside. Odorant receptors (ORs) are expressed on the dendritic membrane of OSNs for reception of the odorants. The external liposoluble odorant molecules penetrate the pores on the sensilla, go into the lymph, are recognized by odorant-binding proteins (OBPs), and delivered to the ORs. The ORs specifically receive the chemicals, transmitting an electrical signal to the brain, and thereby resulting in corresponding behaviors of insects (Clyne et al., 1999; Hallem et al., 2004; Zwiebel and Takken, 2004; Leal, 2013).

Odorant receptors have always been the core of olfactory research because they are essential in the odor recognition process. The first insect OR was identified in *Drosophila melanogaster* (Clyne et al., 1999; Gao and Chess, 1999). Compared with vertebrate ORs, which are G-protein-coupled receptors, insect ORs have the opposite membrane topology, with their N-terminus inside and their C-terminus outside the cell (Benton et al., 2006; Fleischer et al., 2018). The ORs identified in insects can be divided into two types: the first is the non-conventional OR, the olfactory receptor coreceptor (Orco), which is highly conserved and widely expressed among different insects; the second is the conventional OR, which varies widely among various species (Mombaerts, 1999; Benton et al., 2006). It has been widely accepted that insect ORs transduce chemical signals by forming heteromeric complexes with *Orco* (Sato et al., 2008; Grosse-Wilde et al., 2011).

The number of putative insect ORs identified has increased with the progress in sequencing technology and bioinformatics tools, but still varies considerably among insect species. For example, 66 ORs in *Bombyx mori* (Tanaka et al., 2009) and 65 ORs in *Helicoverpa armigera* (Zhang et al., 2015) were identified based on genome and antennal transcriptomic analysis. Moreover, 170 ORs have been found from the genome of *Apis mellifera* (Robertson and Wanner, 2006). The differences in the number of ORs in different insects is assumed to be driven by certain physiological and ecological demands (Fleischer et al., 2018).

As insect ORs continue to be identified, researchers have begun to study the function of ORs at the molecular and cellular levels. Many ORs in several species have been functionally analyzed by heterologous expression systems *in vitro* or *in vivo*, such as *Xenopus* oocyte expression system or the “empty neuron system” in *Drosophila* (Nakagawa et al., 2005; Wanner et al., 2010; Montagné et al., 2012; Liu et al., 2013; Cao et al., 2016; Gonzalez et al., 2016). Using these systems, functional analyses have been carried out for pheromone receptors and other general ORs in several species, including *B. mori* (Sakurai et al., 2004, 2011; Liu et al., 2017), *D. melanogaster* (Hallem et al., 2004, 2006; Kreher et al., 2005), *Spodoptera littoralis* (Fouchier et al., 2017), *H. armigera* (Liu et al., 2013; Cao et al., 2016; Chang et al., 2016; Di et al., 2017), and *H. assulta* (Chang et al., 2016; Cui et al., 2018).

The Asian corn borer, *Ostrinia furnacalis* (Lepidoptera: Crambidae), feeds on various plants including the economic crop maize, causing serious damage and resulting in about 10–30% yield loss of maize in China (Nafus and Schreiner, 1991; Wang et al., 2000). The whole repertoire of the chemosensory genes expressed in the antennae have been identified in *O. furnacalis*, including 54 ORs, 24 OBPs, 19 chemosensory proteins (CSPs), 21 IRs (ionotropic receptors), 5 GRs (gustatory receptors), 2 sensory neuron membrane proteins (SNMPs), and 26 odorant degrading enzymes (ODEs) (Yang et al., 2015). Among them, ORs have been identified as essential for pheromone sensing (Yang et al., 2016). In addition, all the pheromone receptors have been functionally analyzed for understanding the details of pheromone perception in this species (Liu et al., 2018). General ORs should be considered equally important to the pheromone receptors; however, none of them have been studied in *O. furnacalis*.

Nonanal is a plant volatile of maize, which causes a significant electrophysiological response of gas chromatography–electroantennographic detection (GC-EAD) in females of *O. furnacalis* (Zhang et al., 2018). Behavior studies have confirmed that nonanal at a certain concentration has a repellent effect on the oviposition process for female adults in this species. In this study, we identified a female-biased expressed OR that responds to the repellent nonanal using a *Xenopus* oocyte expression system. In addition, we found that OfurOR27 was also sensitive to two other compounds, octanal and 1-octanol, which were confirmed to be repellents in a subsequent behavioral assay. Our results provide two additional repellents for the Asian corn borer by a reverse chemical ecological method and may help to develop new approaches for controlling this pest.

## Materials and Methods

### Insect Rearing

*Ostrinia furnacalis* colonies were maintained at laboratory conditions in the Chinese Academy of Agricultural Sciences, Beijing, China. Insects were reared on an artificial diet at 28°C and kept at 15:9 (L/D) and 80% relative humidity. Male and female adults were fed with 10% sugar solution. Tissues including antennae, proboscis, thorax, legs, and sex glands were dissected from 3-day-old adults, immediately placed in liquid nitrogen, and then stored at −80°C until use.

### Plant Volatile Organic Compounds

A total of 95 odorants from Sigma-Aldrich were used for this experiment (Supplementary Table S1) and were divided into eight groups: pheromone components, green leaf volatiles, terpenoids, aromatics, aldehydes, ketones, alcohols, and esters. All compounds were dissolved in dimethyl sulfoxide (DMSO) at a concentration of 1 M as a stock solution. Before the experiment, the stock solution was diluted to working concentration in 1 × Ringer’s buffer, and 1 × Ringer’s buffer containing 0.1% DMSO was used as a negative control.

### RNA Extraction and cDNA Synthesis

Total RNA from 50 male or female adults was isolated using TRIzol reagent (Invitrogen, Carlsbad, CA, United States) following the manufacturer’s instructions. The RNA was dissolved in RNase-free water, and the integrity was assessed by gel electrophoresis. The concentration and purity of RNA were determined on a NanoDrop ND-2000 spectrophotometer (NanoDrop products, Wilmington, DE, United States). The first-strand complementary DNA (cDNA) was synthesized using the RevertAid First Strand cDNA Synthesis Kit (Fermentas, Vilnius, Lithuania) according to the manufacturer’s instructions.

### Gene Cloning and Expression Vector Construction of *OfurOR27*

The full-length open reading frame (ORF) encoding *OfurOR27* (Acc. No. LC002721) was obtained from antennal transcriptome and was 1,206 bp in length, encoding for 402 amino acids (Yang et al., 2015). The sequence of *OfurOR27* was cloned using the cDNA isolated from antennae, with primers designed by Primer 5.0 (PREMIER Biosoft International, Palo Alto, CA, United States) (Table 1). PrimeSTAR HS DNA polymerase (2 × premix) was used for the PCR reactions (TaKaRa, Dalian, China). A final volume of 50 μl PCR reaction included 25 μl 2 × primerSTAR HS, 2 μl sense and antisense primers (10 mM), 2 μl cDNA, and 19 μl double-distilled H_2_O. The PCR reactions were carried out under the following conditions: incubated at 98°C for 3 min; run with 35 cycles of 98°C for 10 s, 55°C for 15 s, 72°C for 1 min 30 s; and incubated at 72°C for 10 min before being held at 4°C. The amplified PCR products were analyzed on a 1.0% agarose gel and inserted into the cloning vector pEASY-Blunt (TransGene Biotech, Beijing, China) and further sequenced to confirm the sequence. The full-length gene sequence of *OfurOR27* was then ligated into a pT7Ts expression vector using a pair of newly designed primers with *Apa*I and *Xho*I restriction sites (Table 1). The pT7Ts expression vector of *OfurOrco* (*OfurOR2*) was stored at −80°C (Yang et al., 2015). The accession number in DDBJ is LC002697.

**TABLE 1:** Primers used in this study.

**Primers**	**Sequences 5′–3′**	**Purpose**
OfurOR27-F	ATGTCCGACATAACGCTTTC	Gene cloning
OfurOR27-R	TTATTTGTCGTTGTACATAGTGTAA	
OfurOrco-F	ATGATGACCAAAGTGAAAGCTCAG	
OfurOrco-R	CTACTTCAGTTGTACCAAAACCATGA	
OfurOR27F-RT	CGCTAGCAACTATGGAACAGAC	qPCR
OfurOR27R-RT	GGTTCCAGCAAGACAATGGTG	
OfurActinF-RT	CCGTCCTCCTGACCGAGGCTC	
OfurActinR-RT	GGTGTGGGAGACACCATCTCCG	
OfurOR27F-P	TCAGGGCCCGCCACCATGTCCGAC ATAACGCTTTC	cRNA synthesizing
OfurOR27R-P	TCACTCGAGTTATTTGTCGTTGTACAT AGTGTAA	
OfurOrcoF-P	TCAACTAGTGCCACCATGATGACCA AAGTGAAAGCTCAG	
OfurOrcoR-P	TCAGCGGCCGCCTACTTCAGTTGTA CCAAAACCATGA	

### Functional Analysis of OfurOR27 Using a *Xenopus* Oocyte Ectopic Expression System

The expression vector was linearized by restriction enzyme *Sma*I and subsequently used for cRNA synthesis with an mMESSAGE mMACHINE T7 kit (Ambion, Austin, TX, United States). Mature healthy *Xenopus* oocytes (stages V–VII) were incubated with 2 mg/ml collagenase I at pH 7.6 in wash buffer consisting of 96 mM NaCl, 2 mM KCl, 5 mM MgCl_2_, and 5 mM HEPES (pH 7.6) at room temperature for about 1 h until almost all of them were separated (Liu et al., 2013). After being cultured overnight in an incubator at 18°C, the 1:1 mixture of *OfurOR27* and *OfurOrco* cRNA (27.6 ng each) was microinjected into the oocytes. After an incubation for 2–4 days at 18°C in incubation medium (1 × Ringer’s buffer, 5% dialyzed horse serum, 50 mg/ml tetracycline, 100 mg/ml streptomycin, and 550 mg/ml sodium pyruvate), oocytes were connected to a two-electrode voltage clamp, and then, the currents were recorded. Currents induced by odorants were recorded using an OC-725C oocyte clamp (Warner Instruments, Hamden, CT, United States) at a holding potential of −80 mV. Oocytes were exposed to 10^–4^ concentrations of different compounds in a random order for 15 s each, and the interval between exposures allowed the current to return to baseline. Data acquisition and analysis were carried out with Digidata 1440A and Pclamp 10.0 software (Axon Instruments Inc., Union City, CA, United States). At the same time, dose-response data were analyzed using GraphPad Prism 7 (GraphPad Software, San Diego, CA, United States). Statistical comparison of responses to different odors of *OfurOR27* was assessed using GraphPad Prism 7, followed by least-significant difference (LSD) tests.

### Tissue-Specific Expression of *OfurOR27*

To reveal the expression level of *OfurOR27* in different tissues of adults, quantitative polymerase chain reaction (qPCR) was performed using cDNA obtained from antennae (A), proboscis (P), thorax (T), legs (L), and sex glands (SG). *OfurActin* was chosen as the reference gene. The primers are listed in Table 1. GoTaq qPCR Master Mix (Promega, Madison, WI, United States) was used for qPCR, and the reactions were carried out on an Applied Biosystems 7500 Fast Real-Time PCR System (ABI, Carlsbad, CA, United States). The reactions (20 μl) consisted of 10 μl 2 × GoTaq qPCR Master Mix, 1 μl gene primer (10 mM), 1 μl cDNA, and 8 μl RNase-free water. The conditions were 95°C for 2 min; 40 cycles of 95°C for 15 s; and 60°C for 50 s. Each qPCR reaction was performed in triplicate with three separate biological samples to check for reproducibility. The specificity of the primers was measured using a melting curve, and the amplification efficiency was calculated using a standard curve method. *OfurOR27* relative expression levels were analyzed using the relative 2^–ΔCT^ quantitation method, where ΔC_T_ = C_T_ (*OfurOR27*) – C_T_ (*OfurActin*). Statistical comparison of expression of *OfurOR27* was assessed using one-way nested analysis of variance (ANOVA), followed by LSD tests.

### Bioassay of Oviposition in Gravid Female Adults

Behavior analysis for oviposition was carried on for nonanal and other identified candidate odorants. A net cage (25 × 25 × 25 cm) was used with two pieces of plastic wrap (15 × 15 cm) hanging on opposite sides that contained odorants and solvent, respectively. Each odorant was diluted into 100 ng/μl with paraffin oil as the solvent. Fifty gravid females were put into the cage, and after 24 h, eggs laid on the two pieces of plastic wrap were collected and counted under a stereomicroscope (Huang et al., 2009). Three repeats were done for each odorant. The preference of oviposition was calculated as: preference (%) = eggs (odorant)/[eggs (odorants) + eggs (control)], following the methods described in Huang et al. (2009).

### Single-Sensillum Recording (SSR)

Sensilla trichoidea from 2-day-old female adults were used for the recordings. Individuals were fixed in a 1 ml plastic pipette tip and the settings for recording were the same as discribed in Liu et al. (2018). Tungsten wires were used as electrodes, one was inserted into the sensillun (recording electrode) and another was inserted into the opposite eye (reference electrode). The single-sensillum recording (SSR) system was set up with a air pulse controller CS55 and a data acquisition controller IDAC-4 made by Syntech (Kirchzarten, Germany). Recording was performed under a LEICA Z16 APO microscope at 920 × magnification. AutoSpike software (V3.9, Syntech) was used to analyze the data. Odorants at the concentration of 1 μg/μl were used for the recording.

### Phylogenetic Analysis of *OfurOR27*

For the phylogenetic analysis of genes homologous to *OfurOR27*, OR gene repertoires from 14 species in six orders were collected including *O. furnacalis* (Yang et al., 2015), *H. armigera* (Liu et al., 2012; Zhang et al., 2015), *H. assulta* (Zhang et al., 2015), *B. mori* (Wanner et al., 2007), *Chilo suppressalis* (Cao et al., 2014), *Mythimna separata* (Du et al., 2018), *Cydia pomonella* (Walker et al., 2016), *Plutella xylostella* (Yang et al., 2017), *Manduca sexta* (Koenig et al., 2015), *Apis florea* (Karpe et al., 2016), *Locusta migratoria* (Wang et al., 2015), *D. melanogaster* (Clyne et al., 1999; Vosshall et al., 1999; Zwiebel and Takken, 2004), *Apolygus lucorum* (An et al., 2016), and *Acyrthosiphon pisivorum* (Smadja et al., 2009). These species belong to the orders Lepidoptera, Hymenoptera, Orthoptera, Diptera, Hemiptera, and Homoptera. The amino acid sequences were aligned using MAFFT v7.130 (Katoh and Toh, 2010). The phylogenetic tree was constructed and analyzed by the maximum likelihood method using bootstrap replicates with 1,000 × resampling in RAxML version 8 with the Jones–Taylor–Thornton amino acid substitution model (JTT) (Stamatakis, 2014). The transmembrane domains of these genes were predicted using TMHMM version 2.0^1^.

## Results

### OfurOR27 Responds to Nonanal, 1-Octanol, and Octanal

**FIGURE 1:**
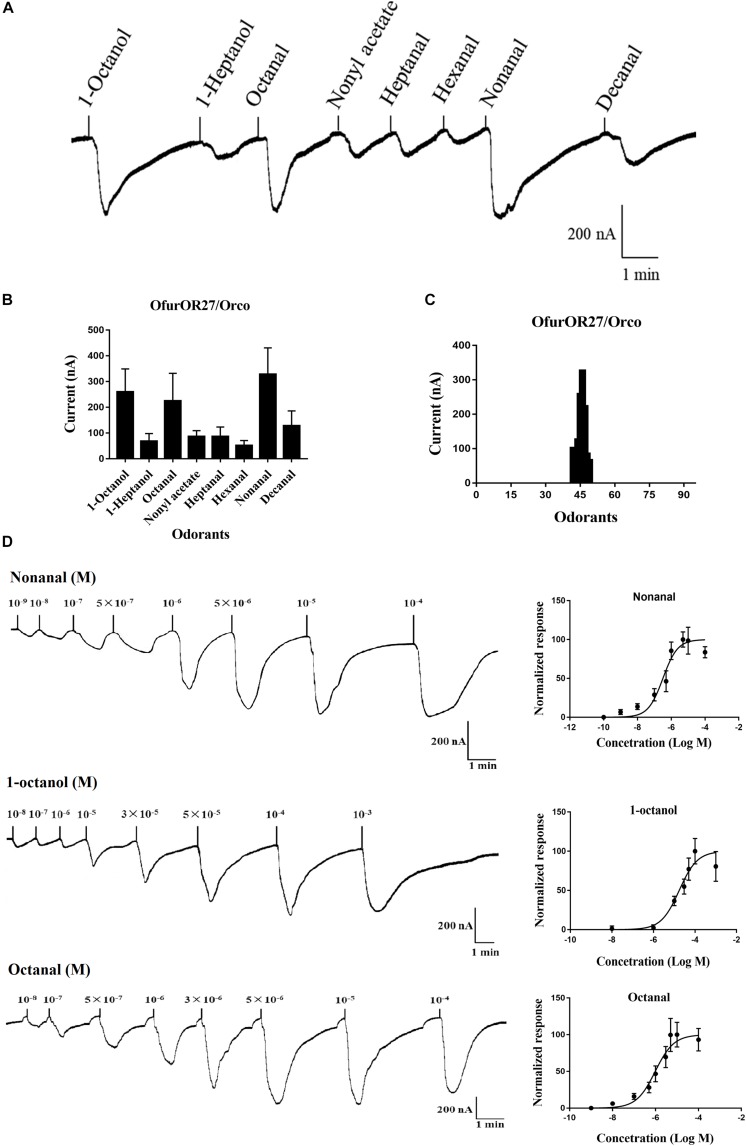
Functional analysis of OfurOR27 using a *Xenopus* oocyte expression system. **(A)** Responses of OfurOR27/Orco to eight identified odorants (10^– 4^ M). **(B)** Response profile of OfurOR27/Orco. Error bars indicate standard error of the mean [*p* < 0.001, ANOVA, least-significant difference (LSD), *n* = 6]. **(C)** Tuning curve for the OfurOR27/Orco to an odorant panel comprising 95 odorants, arranged along the *x*-axis according to the strength of the response they elicit. The odorants that elicited the strongest responses were placed near the center of the distribution, while those that elicited the weakest responses were placed near the edges. **(D)** Dose–response curve for OfurOR27/Orco in responding to nonanal, 1-octanol, and octanal. Error bars indicate SEM (*n* = 6).

In total, 95 compounds were used to identify candidate ligands of OfurOR27 with a two-electrode voltage clamp. OfurOR27 was identified as responding to nonanal but responded to eight chemical substances in total: 1-octanol, 1-heptanol, octanal, nonyl acetate, heptanal, hexanal, nonanal, and decanal (Table 2). Among them, OfurOR27 was most sensitive to nonanal, with responses of about 328.3 ± 41.91 nA. Moreover, OfurOR27 was also sensitive to 1-octanol and octanal, with responses of 260 ± 36.51 nA and 225 ± 43.8 nA, respectively (*p* < 0.01, one-way ANOVA followed LSD test, *n* = 6) (Figure 1). The dose–response study confirmed the sensitivity of OfurOR27 to nonanal, 1-octanol, and octanal, with the EC50 value of 3.673 × 10^–7^ M, 1.406 × 10^–5^ M, and 1.184 × 10^–6^ M, respectively (Figure 1D). Response values to 1-heptanol, nonyl acetate, heptanal, hexanal, and decanal were not as high, at only ∼100 nA (Figures 1A–C).

**TABLE 2:** Ligands identified for OfurOR27/Orco.

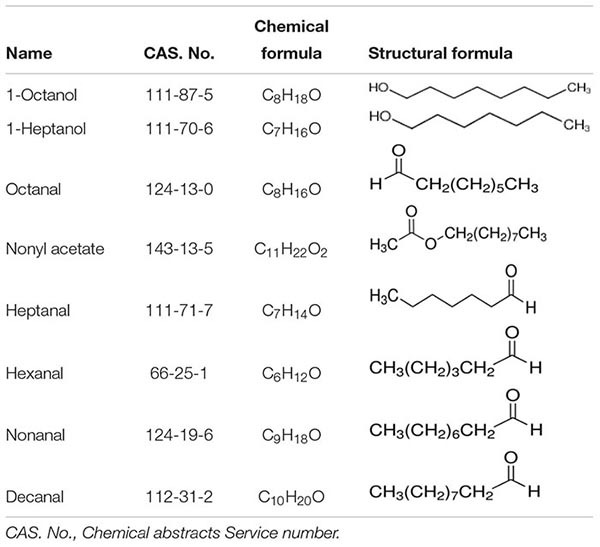

### Tissue Expression Profiles of OfurOR27

**FIGURE 2:**
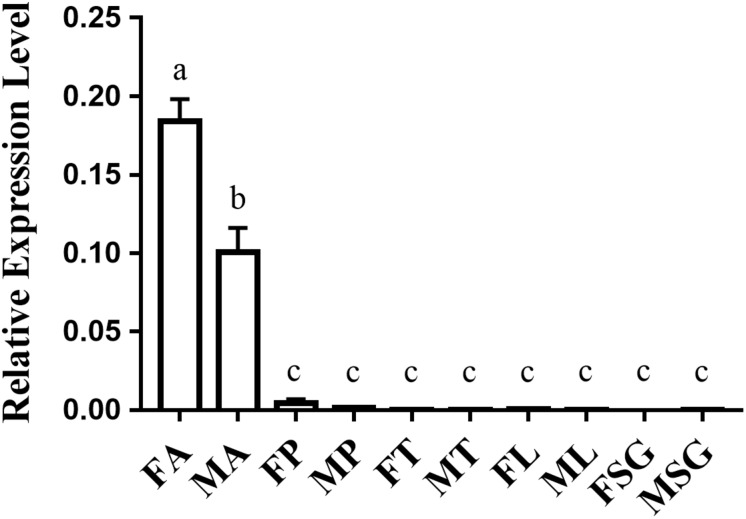
Tissue-specific expression of *OfurOR27* in *O. furnacalis*. FA, female antennae; MA, male antennae; FP, female proboscis; MP, male proboscis; FT, female thorax; MT, male thorax; FL, female legs; ML, male legs; FSG, female sex glands; MSG, male sex glands. Error bars represent the standard error; those labeled with different letters are significantly different [*p* < 0.05, ANOVA, least-significant difference (LSD)].

The tissue-specific expression analysis indicated that *OfurOR27* was predominantly expressed in the antennae of adults, with relative expression levels of more than 0.1, compared to those in proboscis, thorax, legs, and sex glands, with the relative expression levels of 0.00291, 0.00192, 0.00223, and 0.00255, respectively (Figure 2). Moreover, *OfurOR27* showed female-biased expression in the antennae, which was consistent with a previous study (Yang et al., 2015).

### Nonanal, 1-Octanol, and Octanal Are Repellents for *O. furnacalis*

**FIGURE 3:**
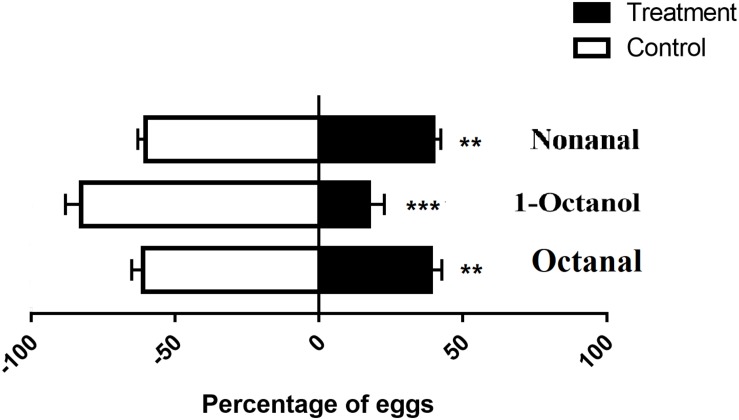
Oviposition assay using three repellents for gravid females in *O. furnacalis*. The black bars indicate the preference of oviposition for each test chemical. The asterisks indicate a significant difference (**p < 0.01; ***p < 0.001).

*Ostrinia furnacalis* females laid fewer eggs on the plastic containing nonanal. This result was consistent with a previous study (Zhang et al., 2018). In addition, we identified that 1-octanol and octanal also had a repellent effect causing females to avoid laying eggs while these odorants were present. The preference rates of nonanal, 1-octanol, and octanal were 39.7, 17.3, and 38.8%, respectively (Figure 3). Among them, 1-octanol displayed a better effect as a repellent to oviposition for *O. furnacalis* females.

### Single-Sensillum Recording for Nonanal, 1-Octanol, and Octanal

Sensilla trichoidea were used for SSR in *O. furnacalis*. Most of the recorded sensilla did not respond to nonanal, 1-octanol, or octanal. Three types of sensilla responded to the odorants (Figure 6). Type A is a short sensilla trichoidea mainly distributed on the side of the female antennae, while types B and C are long sensilla trichoidea at the center of the female antennae. Type C responded to nonanal, 1-octanol, and octanal. Type A responded to 1-octanol and octanal. Type B seemed to only respond to nonanal, but we only recorded it once, possibly due to interference by insect movement.

### Phylogenetic Analysis of Homologous Genes of *OfurOR27*

**FIGURE 4:**
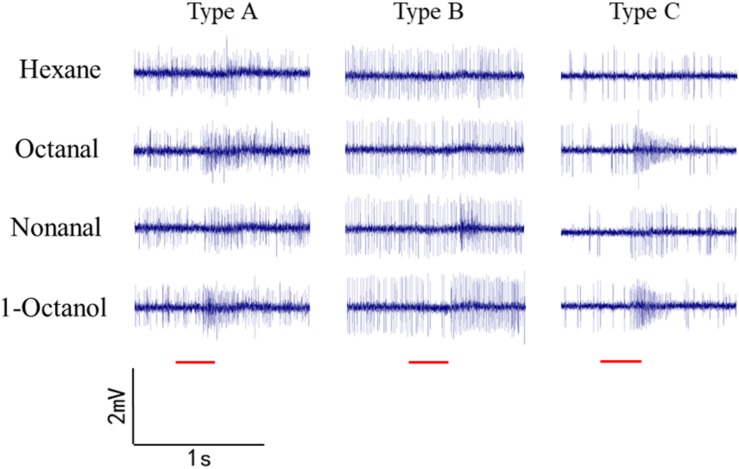
Single sensillum recordings of *s. trichoidea* from female adults in *O. furnacalis*. Three different types (Type A–C) of *s. trichoidea* characterized by the response to nonanal, 1-octanol and octanal. The stimulus was applied for 300 ms which was represented with a red line under the trace.

**FIGURE 5:**
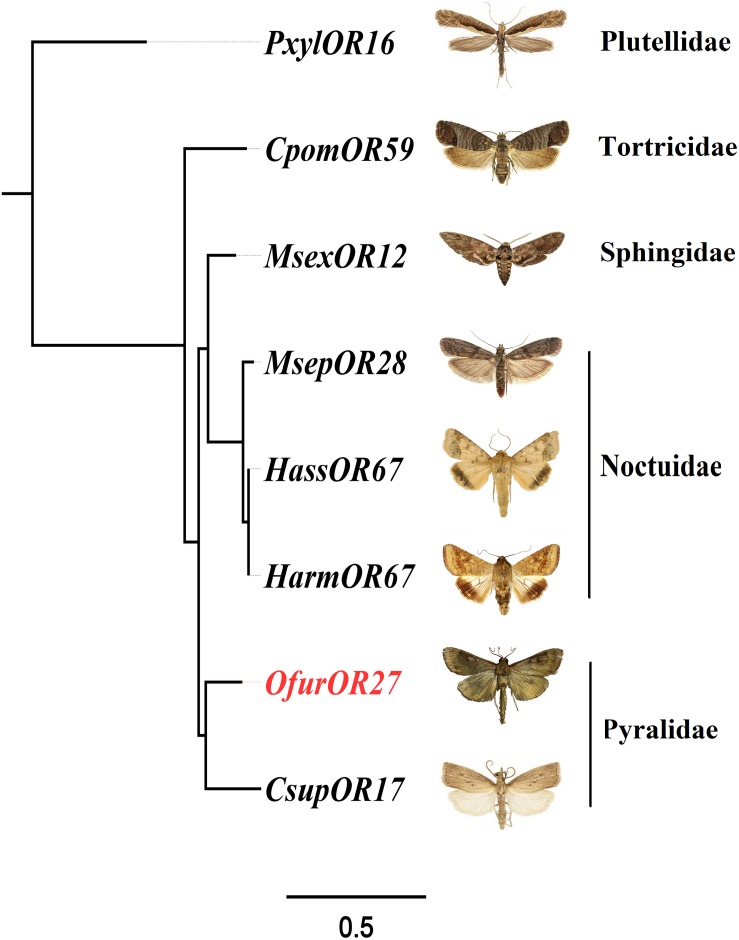
Phylogenetic analysis for *OfurOR27* and its homologous genes. Odorant receptors including *PxylOR16*, *MsexOR12*, *MsepOR28*, *HassOR67*, *HarmOR67*, *CsupOR17*, and *CpomOR59* (Supplementary Dataset S1) were downloaded from the National Center for Biotechnology Information. All sequences were aligned using MAFFT software. The phylogenetic analysis was conducted by RAxML version 8. The final tree was visualized by FigTree version 1.4.0 software (http://tree.bio.ed.ac.uk/software/figtree/).

To identify homologous genes of *OfurOR27*, phylogenetic analysis was carried out with 1,075 ORs from 14 species. Seven ORs clustered into the same clade with *OfurOR27*, including *PxylOR16*, *MsexOR12*, *MsepOR28*, *HassOR67*, *HarmOR67*, *CsupOR17*, and *CpomOR59* from five families in Lepidoptera (Figure 4). Homologous genes were only found in lepidoptera insects, but no homologous genes were found in *B. mori*. We speculated that degradation may have occurred in *B. mori*. All the homologous genes were aligned with *OfurOR27*. The correlation prediction software was used to predict the number of transmembrane structures of seven ORs, and the results showed that they all have seven transmembrane domains and the N-terminus was located within the cell membrane (Figure 5). Sequence alignment of *OfurOR27* with its seven orthologous genes showed that they shared 77.56% amino acid identity. Moreover, *PxylOR16*, *MsexOR12*, *MsepOR28*, *HassOR67*, *HarmOR67*, *CsupOR17*, and *CpomOR59* were aligned with *OfurOR27* and found to have 56.67, 73.81, 69.29, 70, 70, 72.62, and 69.52% identity, respectively.

**FIGURE 6:**
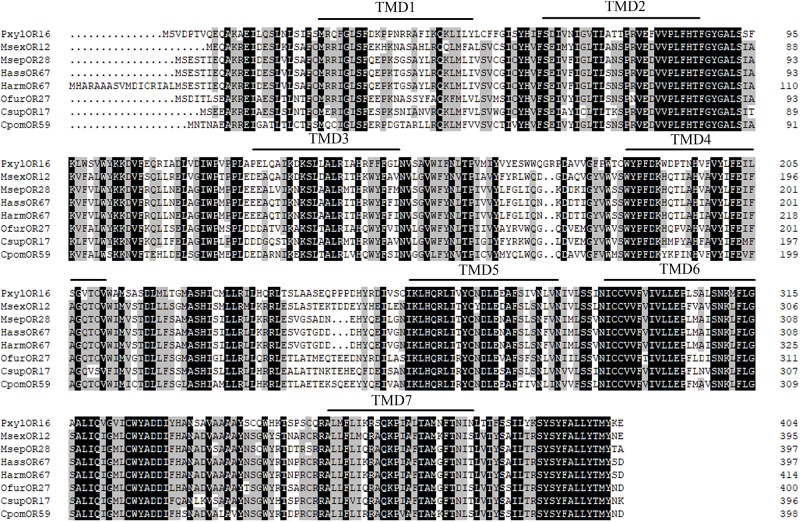
Alignment of the amino acid sequences of *OfurOR27* and its homologous genes. The transmembrane domains of genes were predicted using TMHMM version 2.0 (http://www.cbs.dtu.dk/services/TMHMM/), and sequences were aligned using DNAMAN version 8 software (Lynnon LLC, San Ramon, CA, United States). Amino acids identical in all sequences were marked with black shading. Numbers to the left refer to the position of the last residue in a line in each odorant receptor (OR) sequence. The horizontal lines indicated the position of predicted transmembrane domains.

## Discussion

Use of chemical pesticides is still the primary approach for controlling pests. Integrated pest management (IPM) has been strongly advocated in modern agriculture. The new approach of pest management using chemical ecology is an important part of IPM. Attractants such as pheromones have been applied controlling various pests due to a high degree of commercialization. For repellents, only a few compounds have been made as commercial products. The most famous odorant is *N*,*N*-diethyl-m-toluamide (DEET), which was an effective broad-spectrum mosquito repellent (Bressac and Chevrier, 1998). However, most of these repellents were identified for controlling pests of human health concern such as mosquitos and cockroaches (Li and Boyaro, 2002). For agricultural pests, the “pull and push” strategy was favored. In this strategy, researchers combined attractants and repellents together to control pests (Miller and Cowles, 1990). Recently, herbivore-induced plant volatiles were identified to mediate tritrophic interactions (Turlings and Erb, 2018). This research opened a new approach for pest management through chemical ecology.

Olfaction allows insects to distinguish chemical signals to complete a series of behaviors, such as locating food, sexual partners, and oviposition sites. To successfully perform these behaviors, the sensitive olfactory system of insects must respond to chemical stimuli at the appropriate time (Gadenne et al., 2016). Previous studies have shown that some general ORs may be involved in the common behaviors in both males and females (Zhang et al., 2013), whereas other ORs with biased expression in females or males may be involved in sex-specific behaviors (Anderson et al., 2009; Liu et al., 2014; Yan et al., 2015). Plant volatiles such as linalool, benzoic acid, and 2-phenylethanol have been used as oviposition clues for some female moths (Røstelien et al., 2005; Anderson et al., 2009). Some ORs are functionally characterized as receptors to these key compounds (Anderson et al., 2009; Jordan et al., 2009). OR4 from domestic mosquitoes selectively responds to the released odor of humans and functions directly in host identification and blood sucking (McBride et al., 2014). *AlucOR46* might be related to locate the host plants of *Apolygus lucorum* (Zhang et al., 2016). However, many other ORs are still orphans, and we lack overall awareness of how insects detect plant volatiles.

In this study, we identified an OR with female-biased expression that responds to the repellent nonanal using a *Xenopus* oocyte expression system. In addition, we found that OfurOR27 was also sensitive to two other compounds, octanal and 1-octanol, which were confirmed to be repellents by a subsequent behavioral assay. Single-sensillum recordings were conducted for nonanal, octanal, and 1-octanol, and indicated that *OfurOR27* may be expressed on the sensilla trichoidea. Octanal is a plant green leaf compound, which was confirmed to significantly repel mosquitoes (Logan et al., 2010). Moreover, 1-octanol, an aroma component of tea, may not be a repellent for pest, but it is an attractant for natural enemies *Sphaerophoria menthastri* and *Chrysopa pallens* in tea gardens (Han and Zhou, 2004). Phylogenetic analysis showed that some pest species have genes homologous to *OfurOR27*, which might indicate that they have similar functions and that all three repellents might be applied in other species.

Above all, our results indicated that OfurOR27 is one of the corresponding ORs for nonanal, octanal, and 1-octanol, which all negatively affect *O. furnacalis*. A further study should be carried out to identify other ORs that respond to the three repellents to clarify the molecular mechanism of chemosensation for each odorant. Using reverse chemical ecology, we determined that OfurOR27 is a common receptor for repellents and found two additional repellents for *O. furnacalis*, which may contribute to developing environment-friendly approaches to control this maize pest.

## Data Availability Statement

The data relating to OfurOR27 (LC002721) may be accessed from the DDBJ website: http://getentry.ddbj.nig.ac.jp/getentry/na/LC002721/?format=flatfile&filetype=html&trace=true&show_suppressed=false&limit=10.

## Author Contributions

GW and BY designed the experiments. JY, YC, and YZ carried out the experiments. JY and BY analyzed the experimental results. JY and BY wrote the manuscript.

## Conflict of Interest

The authors declare that the research was conducted in the absence of any commercial or financial relationships that could be construed as a potential conflict of interest.

## Footnotes

1 http://www.cbs.dtu.dk/services/TMHMM/
