# Supplementary material for: Identification of a General Odorant Receptor for Repellents in the Asian Corn Borer Ostrinia furnacalis
Source: Front Physiol. 2020 Mar 13;11:176. doi: 10.3389/fphys.2020.00176 (PMC7083148; doi:10.3389/fphys.2020.00176)
Supplement: Supplementary file 1 [file Data_Sheet_1.pdf]

Table S1 Odorants used in this study.

Number	Odorant	CAS. No.
1	Z12-14AC	35153-20-9
2	E12-14AC	35153-21-0
3	E11-14OH	35153-18-5
4	Z11-14AC	20711-10-8
5	Z9-14AC	16725-53-4
6	(Z)-3-Hexene-1-ol	928-96-1
7	(Z)-2-Hexen-1-ol	928-94-9
8	1-Hexanol	111-27-3
9	(E)-3-Hexen-1-ol	928-97-2
10	1-Hexen-3-ol	4798-44-1
11	(E)-2-Hexen-1-ol	928-95-0
12	2-Ethyl-1-hexanol	104-76-7
13	3-Hexanol	623-37-0
14	2-Hexanol	626-93-7
15	(E)-2-Hexenal	6728-26-3
16	(E)-2-Hexenyl acetate	2497-18-9
17	Geraniol	106-24-1
18	1S-(-)-Verbenone	1196-01-6
19	(-)-Borneol	464-45-9
20	(-)-(E)-Pinocarveol	547-61-5

21	(-)-Linalool	126-91-0
22	Linalool	78-70-6
23	(+)-Cedrol	77-53-2
24	(1S)-(+)-(Z)-Verbenol	112-92-5
25	α-Terpineol	98-55-5
26	Myrcene	123-35-3
27	(R)-(+)-Limonene	5989-27-5
28	α- Pinene	80-56-8
29	(-)-β-Pinene	18172-67-3
30	(S)-(-)-Limonene	5989-54-8
31	Farnesene，mixture of isom	502-61-4
32	Ocimene	13877-91-3
33	Cumene	98-82-8
34	Sabinene	3387-41-5
35	β-Caryophyllene	87-44-5
36	Methyl jasmonate	39924-52-2
37	Geranyl acetate	105-87-3
38	β-Ionone	14901-07-6
39	(S)-(+)-Carvone	2244-16-8
40	(1R)-(-)-Myrtenal	18486-69-6
41	(±)-Citronellal	106-23-0
42	Hexanal	66-25-1

43	Nonyl acetate	143-13-5
44	Decanal	112-31-2
45	1-octanol	111-87-5
46	Nonanal	124-19-6
47	Octanal	124-13-0
48	Heptanal	111-71-7
49	1-Heptanol	111-70-6
50	Cinnamaldehyde	104-55-2
51	2-Phenylethanol	60-12-8
52	Benzyl alchol	100-51-6
53	Methyl benzoate	93-58-3
54	Methyl salicylate	119-36-8
55	Ethyl benzoate	93-89-0
56	Methyl 2-Methoxy benzoate	606-45-1
57	Butyl salicylate	2052-14-4
58	Methyl phenylacetate	101-41-7
59	4'-Ethylacetophenone	937-30-4
60	4-Ethylbenzaldehyde	4748-78-1
61	3-Vinylbenzaldehyde	19955-99-8
62	Benzaldehyde	100-52-7
63	Phenylacetaldehyde	122-78-1
64	Salicylaldehyde	90-02-8

65	p-Anisaldehyde	123-11-5
66	1,4-Diethylbenzene	105-05-5
67	2,6-Di-tert-butylphenol	128-39-2
68	Tridecanal	10486-19-8
69	Undecanal	112-44-7
70	Dodecanal	112-54-9
71	2-Undecanone	112-12-9
72	2-Hexanone	591-78-6
73	2-Heptanone	110-43-0
74	3-Hexanone	589-38-8
75	4-Heptanone	123-19-3
76	4-Nonanone	4485-09-0
77	2-Nonanone	821-55-6
78	3,7-Dimethyl-3-octanol	78-69-3
79	4-hydroxy-4-methyl-2pentanone	123-42-2
80	Tetradecanol 14:OH	112-72-1
81	1-Octen-3-ol	3391-86-4
82	Octadecanol	112-92-5
83	4-Heptanol	589-55-9
84	3-Methyl-1-butanol	123-51-3
85	(Z)-3-Hexenyl acetate	3681-71-8
86	Heptyl butyrate	5870-93-9

87	Hexyl acetate	142-92-7
88	Benzyl acetate	140-11-4
89	Ethyl hexanoate	123-66-0
90	Allyl Isothiocyanate	1476-23-9
91	Hexyl hexanoate	6378-65-0
92	Amyl acetate	628-63-7
93	Amyl butyrate	540-18-1
94	Octyl butyrate	110-39-4
95	2-Propenoic acid butyl ester	141-32-2

Dataset 1 Sequences used for phylogenetic analysis in this study.

>PxylOR16

MSVDPTVQEQAKAEILQSLNLSIFSMRQFGLSFDKPPNRRAFIKQKLILYLCFFGISYHIFSD

IVNIGVTLATTPRVEFVVPLFHTFGYGALSSFKLWSVWYKKDVFEQRIADLVDIWPVPPLA

PELQAIKDKSLLALRIAHRFFFGLNVSAVWIFNLTPVMIYVYESWWQGRPDAVVGFPWTC

WYPFDKWDPTNHVFVYLFEILSGVTCVWAMSASDLMLTGMASHICMLLRILHQRLTSLA

ASEQPPPDHYRDIVSCIKLHQRLIVYCNDLEEAFSIVNLVNIVLSSINICCVVFVIVLLEPLSA

LSNKMFLGAALIQVGVICWYADDIYHANSAVAAAAYSCQWHKTSPSCQRALMFLIKRSQ

KPIALTAMNFTNINLTTFSSILYKSYSYFALLYTMYKEN

>MsexOR12

MEQAKREIDESLKLSAFCMRRIGLSFEKHKNASAHLRQQLMFALSVCSICYHVFSEIMYIG

LTLANSPRVEDVVPLFHTFGYGALSIAKVFALWYKKDVFSQLLRELVGIWPTPPLEDEAQA

IKDKSLDALRITHKWYFAVNVLGVWFYNVTPIAVYFYRLWQDGDAQVGYVWVSWYPFD

KHQTIAHVAVYIFEIFAGQTCVWIMVSTDLLLSGMASHISMLLRMLKRRLESLASTEKTDD

EYYHEILENIKLHQRLITYCYDLEDAFSLSNLVNIVLSSLNICCVVFVIVLLEPFVAVSNKLF

LGSALIQIGMLCWYADDIFHANADVAAAAYNSGWYSTNARCRRALLFLMQRAQKPIAFT

AMKFTNISLVTYSAILTRSYSYFALLYTMYNEN

>MsepOR28

MSESTIEQAKREIDESLVLSAFCMRRIGLSFQEPKSGSAYLRQKLMLIVSVCGICYHVFSEIV

FIGLTLANSPRVEDVVPLFHTFGYGALSIAKVFVLWYKKDVFRQLLNELAGIWPMPPLEEE

ALVIKNKSLAALRMTHRWYFFMNVSGVWFYNLTPIVVYLFGLIQGKDDKIGYVWSSWYP

FDKHQTLAHVAVYLFEIFAGQTCVWIMVSTDLLFSAMASHISILLRLLKRRLESVGSADNE

HYQEILGNIKLHQRLITYCNDLENAFSLSNFVNIMLSSVNICCVVFVIVLLEPLMAISNKLF

LGSALIQIGMLCWYADDIFHANADVSAAAYNSCWYRTDTRSRRALLFLIQRAQKPIAFTA

MGFTNITLVTYSAILTRSYSYFALLYTMYTA

>HassOR67

MSESTIEQAKREIDESLVLSAFCMRRIGLSFQEPKTGSAYLRQKLMLIVSVCGICYHVFSEIV

FIGLTLSNSPRVEDVVPLFHTFGYGALSIAKVFVLWYKKDVFRQLLNELAGIWPMPPLEEE

AQTIKNKSLAALRITHRWYFFINVSGVWFYNLTPIVVYLFGLIQNKDDTIGYVWSSWYPFD

KHQTLAHIAVYLFEIFAGQTCVWIMVSTDLLFSAMASHISILLRLLKRRLESVGTGDDDHY

QEIVGNIKLHQRLITYCNDLENAFSLSNFVNIMLSSVNICCVVFVIVLLEPLMAISNKLFLGS

ALIQIGMLCWYADDIFHANADVAAAAYNSGWYRTNPRCRRALLFLIKRAQKPIAFTAMGF

TNITLVTYSAILTRSYSYFALLYTMYSDD

>HarmOR67

MHARAAASVMDICRIALMSESTIEQAKREIDESLVLSAFCMRRIGLSFQEPKTGSAYLRQK

LMLIVSVCGICYHVFSEIVFIGLTLSNSPRVEDVVPLFHTFGYGALSIAKVFVLWYKKDVFR

QLLNELAGIWPMPPLEEEAQTIKNKSLAALRITHRWYFFINVSGVWFYNLTPIVVYLFGLI

QNKDDTIGYVWSSWYPFDKHQTLAHIAVYLFEIFAGQTCVWIMVSTDLLFSAMASHISILL

RLLKRRLESVGTGDDDHYQEIVGNIKLHQRLITYCNDLENAFSLSNFVNIMLSSVNICCVV

FVIVLLEPLMAISNKLFLGSALIQIGMLCWYADDIFHANADVAAAAYNSGWYRTNPRCRR

ALLFLIKRAQKPIAFTAMGFTNITLVTYSAILTRSYSYFALLYTMYSDD

>OfurOR27

MSDITLSEAKREIAESLTLNTFCMRRIGLSFEEPKNASSYFAQKFMLVLSVMSICYHVFSEIV

YIGLTLSNSPNVEDVVPLFHTFGYGALSIAKVFALWYKKDVFKQLISELAGIWPMSPLDDD

ATVIKAKSLTALRIAHQWYFVINVLGVWFYNLTPIIVYAYRVWQGQDVEMGYVWVSWYP

FDKHQPVAHVAVYIFEIFAGQTCVWIMVGTDLLFSGMASHIGLLLRLLQRRLETLATMEQT

EEDNYRDILASIKLHQRLIRYCNDLEVAFSFSNLVNIILSSVNICCVVFTIVLLEPFLDISNKL

FLGSALIQIGMLCWYADDILHANADVAAAAYTSGWYRTSARCRRALLFLIQRAQKPIAFT

AMGFTDISLVTYSSILTRSYSYFALLYTMYNDK

>CsupOR17

MSEEAKREIAESLSLNTFCMERIGISFESPKSNIANVRQKLMFVLSVWGICYHVFSEIAYICL

TLTKSPRVEDVVPLFHTFGYGALSITKLFVLWYKKNVFKQLIFELAGIWPLPPLDDDGQST

KNKSLAALRMTHRWYFAVNVLGVWFYNLTPIGIYFYRKWQGLDVEMGYVWVSWYPFD

KHMPYAHFAVYIFEMFAGQVSVFIMVSTDLLFSSMASHISLLLRLLHRRLEALATTNKTEH

EQFDEISANIKLHQRLIRYCNDLESAFSLSNLVNVVLSSINICCVVFVIVLLEPFLNVSNKLF

LGSALIQIGMLCWYADDIFQANLKVSAAAYNSGWYHTSPRCRRAILFVIQRAQKPIAFTA

MGFTNITLVTYSAILTRSYSYFALLYTMYNKG

>CpomOR59

MNTNAEARREIGATLTLCTFSMQCIGLSFERPDGTARLLRQKLMFVVSVCTIVYHVFSEIV

YIGLTLSNSPRVEDVVPLFHTFGYGALSIAKVFALWSKKNVFTEHLDELSGIWPMEPLDED

ARNIKEKSLTALRLVHQWYFSINVGGVLFYNVTPICVYMYQLWQGQDAVVGFVWMSWY

PFDKYKPINHVFVYIFEVFAGQTCVWIMICTDLLFSGLASHIAMLLRLLHKRLETLAETEKS

QEEYYQEIVANIKLHQRLIRYCNDLEEAFTIVNLINVVFSSLNICCVVFVIVLLEPFMAVSNK

LFLGSALIQIGMLCWYADDIFHSNADVALAVYNSGWYRTDPRCRRALIFLIRRAQKPVAFT

AMKFTNLSLVTYSSILTRSYSYFALLYTMYNDS
